# Correlations between Apparent Diffusion Coefficient (ADC) and Prognosis in Patients with Locally Advanced Rectal Cancer

**DOI:** 10.3390/life14101282

**Published:** 2024-10-10

**Authors:** Silvia Girolama Drago, Cesare Maino, Teresa Paola Giandola, Paolo Niccolò Franco, Rocco Corso, Cammillo Talei Franzesi, Anna Pecorelli, Davide Ippolito

**Affiliations:** 1 Department of Diagnostic Radiology, IRCCS Fondazione San Gerardo dei Tintori, Via Pergolesi 33, 20900 Monza, MB, Italy; sgd.drago@gmail.com (S.G.D.); mainocesare@gmail.com (C.M.); teresagiandola1990@gmail.com (T.P.G.); francopaoloniccolo@gmail.com (P.N.F.); rocco.corso@irccs-sangerardo.it (R.C.); ctfdoc@gmail.com (C.T.F.); davide.ippolito@unimib.it (D.I.); 2 Radiologia Addomino Pelvica Diagnostica e Interventistica IRCCS Azienda Ospedaliera Universitaria di Bologna Policlinico di Sant’Orsola, Via Pietro Albertoni 15, 40138 Bolonga, BO, Italy; 3 School of Medicine, University of Milano Bicocca, Via Cadore 33, 20090 Monza, MB, Italy

**Keywords:** magnetic resonance imaging, neoplasms, rectal, diffusion magnetic resonance imaging

## Abstract

Background: the aim of this study is to assess the performance of diffusion-weighted imaging (DWI) and apparent diffusion coefficient (ADC) values in predicting the response to neoadjuvant chemoradiation therapy (CRT) and outcome in patients with locally advanced rectal cancer (LARC). Materials and Methods: ninety-four patients with magnetic resonance imaging (MRI) pre- and post-neoadjuvant treatment were retrospectively enrolled. Three regions of interest (ROIs) were manually drawn on three different slices of the tumor for every DWI sequence. ROIs were positioned to include only high signal areas and avoid artifacts or necrotic areas. ROIs were automatically copied onto the corresponding ADC maps and the system derived three different ADC values, distinguishing between mean, maximum, and minimum values, and the standard deviation (SD). Only mean ADC values were considered. After surgical intervention, pTNM and the Mandard tumor regression grade (TRG) were obtained. Patients with a TRG of 1–2 were classified as responders, while patients with a TRG from 3 to 5 were classified as non-responders. Results: no correlation was found between pre-ADC values and TRG classes, while post-ADC and ΔADC values showed a significant correlation with TRG classes (r = −0.285, *p* = 0.002 and r = −0.290, *p* = 0.019, respectively). Post-ADC values were statistically different between responders and non-responders (*p* = 0.019). When considering the relation between overall survival (OS) and ADC values, pre-ADC showed a negative correlation with OS (r = −0.381, *p* = 0.001), while a positive correlation was found between ΔADC values and OS (r = 0.323, *p* = 0.013). According to ΔADC values, the mean OS time between responders and non-responders showed a significant difference (*p* = 0.030). A statistical difference was found between TRG classes and OS (*p* = 0.038) and by dividing patients in responders and non-responders (*p* = 0.019). Conclusions: the pre-ADC and ΔADC values could be used as useful predictors for patient prognosis, thus helping the treatment planning. On the other hand, the post-ADC values, thanks to their relationship with the TRG classes, could be the ideal tool to predict the histopathological response and plan a conservative approach to the treatment of rectal cancer.

## 1. Introduction

Rectal cancer is the third most common malignancy diagnosed in the Western society [1,2]. Nowadays, magnetic resonance imaging (MRI), particularly thanks to diffusion-weighted techniques (DWI), allows to improve tissue differentiation and characterization according to lesions cellularity [3] and is considered the reference standard for staging and restaging rectal cancer patients. MRI has gained this central role due to different advantages, including its non-invasiveness, the high contrast resolution, and the wide availability. On these bases, MR is extremely useful during the pre-operative stages, for monitoring tumor response, detecting recurrence, and, finally, for evaluating node metastases. Particularly, DWI can help identify rectal tumors by showing areas of restricted diffusion that correlate with the presence of malignant cells. Moreover, DWI can be used to monitor treatment response in rectal cancer patients undergoing neoadjuvant chemoradiotherapy (CRT) [4]: changes in diffusion characteristics can indicate a treatment response before visible changes occur on standard imaging.

Finding a precise correlation between apparent diffusion coefficient (ADC) values and pathological responses could help define which patients can be subjected to less invasive surgical approaches. In this field, the watch-and-wait (WW) approach is attracting growing interest in the medical community. According to Habr-Gama’s study group [5], patients who show a complete response to CRT may avoid surgical intervention, while their prognosis does not significantly differ from those who are candidates for surgery [5,6].

Regarding the responses to CRT, one of the most widely accepted scoring systems is the Mandard tumor regression grade (TRG). The score categorizes the response into five grades, specifically 1 for complete response, 2 for near complete, 3 for moderate, 4 for mild, and 5 for no response [7]. However, Mandard’s classification is based on biopsy specimens, an approach which is not pitfall-free. On the other hand, considering the abovementioned advantages of MRI with respect to restaging patients with locally advanced rectal cancer (LARC), it is of the utmost importance that we explore its potential as a prognostic tool for detecting progression or relapse [8,9,10].

On these bases, our retrospective study aims to evaluate the potential role of ADC values as a predictive tool in patients with LARC, using histopathological correlation as the reference standard.

## 2. Materials and Methods

Upon reviewing the protocol, the local ethical committee deemed formal approval unnecessary, owing to the retrospective, observational, and anonymous nature of this study.

### 2.1. Data Collection

Patients were selected according to the following inclusion criteria: (1) biopsy-proven rectal adenocarcinoma, (2) staging MR examination before CRT, (3) restaging MR examination after completing CRT.

The exclusion criteria were: (1) histotype different than adenocarcinoma, (2) non-LARC patients, (3) surgery performed without previous neoadjuvant therapy, (4) MR performed with suboptimal, or without, DWI sequences, (5) patients with clinical or technical contraindication to MRI (e.g., non-compatible bio metallic implants or claustrophobia), (6) insufficient image quality due to the presence of artifacts (e.g., in case of metallic prothesis).

The response rate was assessed by distinguishing between responders and non-responders: patients in TRG classes 1 and 2 were considered responders, while those in TRG 3, 4, and 5 were regarded as non-responders. Finally, for each patient, we registered the overall survival time in months (OS).

### 2.2. MRI Protocol

MR examinations were performed before and after the CRT neoadjuvant treatment. All the examinations were performed on a 1.5 T unit scanner (Achieva Plus; Philips, The Netherlands) with a surface coil.

The protocol included: wide FOV axial and sagittal TSE T2W, axial TSE T1W, and para-axial and para-coronal TSE T2W, orthogonal and parallel to the long axis of the tumor, respectively.

An axial DWI with fat signal suppression was acquired, with *b* values of 0 s/mm^2^ and 1000 s/mm^2^. All technical parameters are summarized in Table 1.

No dynamic fat-saturated T1W sequences before and after the contrast medium injection were considered in the standard protocol, as suggested in the literature [3].

No intestinal cleansing nor spasmolytic drugs were administered. Distension of the rectal lumen was attained through the administration of a small amount (100 mL) of ultrasound gel.

### 2.3. MR Imaging Analysis

A single radiologist with more than 15 years of experience in rectal MRI, blinded with respect to clinical and pathology data, analyzed the T2W and DWI images to correctly diagnose and localize the primary rectal cancer during the staging phase and after CRT. The rectal tumor was classified as a hypointense lesion compared to the surrounding fat tissue and as slightly hyperintense compared to the pelvic muscles. The mesorectal fascia (MRF) was identified as a tiny hypointense line surrounding the mesorectal fat.

ADC maps were automatically generated from the native DWI sequence by using a dedicated imaging workstation (Philips Intellispace Portal 10.0—Philips Healthcare).

Three regions of interest (ROIs) were manually drawn on three different slices of the tumor for every DWI sequence. ROIs were positioned to include only high signal areas and avoid the inclusion of artifacts or necrotic areas, if present. ROIs were automatically copied onto the corresponding ADC maps, and the system derived three different ADC values, distinguishing between mean, maximum, and minimum values, and the standard deviation (SD). Only mean ADC values were considered. ADC values were measured during staging MRI (pre-ADC) and during MR examination after CRT (post-ADC). Finally, ΔADC was calculated as the difference between pre- and post-ADC values for every single patient, according to the formula: (ADC_post_ − ADC_pre_)/ADC_pre_.

In case the primary rectal cancer could not be assessed after CRT, ROIs were drawn on the residual fibrotic tissue visible on the T2W images or on the bowel wall, where the tumor was observed on the staging images.

### 2.4. Statistical Analysis

Continuous variables were expressed as means and standard deviations and compared by using the Mann–Whitney U test or the Wilcoxon rank test, as appropriate. Categorical variables, expressed as numbers and percentages, were compared by using the χ^2^ test. Correlations were assessed using the Spearman’s correlation coefficient, when appropriate. Receiver operating characteristic (ROC) curves were designed to define the capability of ADC values to assess treatment responses.

All tests were two-sided, and *p* < 0.05 was considered statistically significant. All statistical analyses were performed using the SPSS statistical package software (version 26.0; SPSS, Chicago, IL, USA).

## 3. Results

### 3.1. Patient Population and TRG Classes

**Figure 1:**
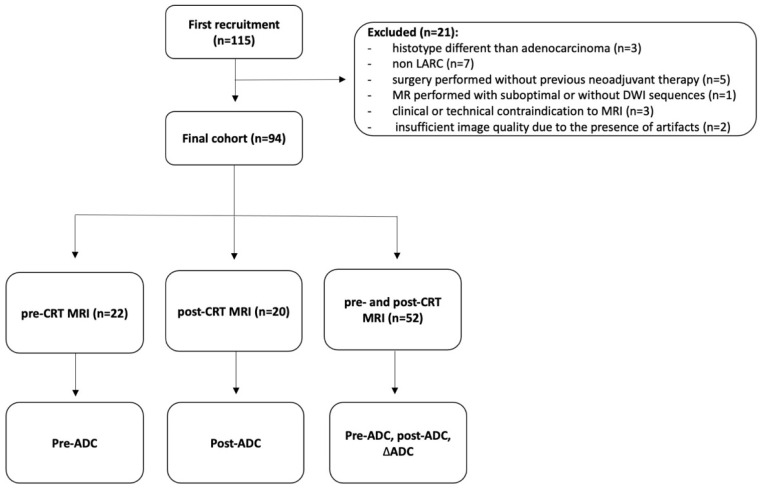
Flow chart of the study.

MRI examinations of 115 patients who underwent staging and restaging for rectal cancer between 2006 and 2018 were analyzed. By applying both inclusion and exclusion criteria, 94 patients were ultimately included in the present study (33 F, 61 M; mean age 64.7 years), resulting in three different groups. The first group was composed of 52 patients (55.3%) with pre- and post-CRT MR examinations, allowing for the evaluation of pre-ADC, post-ADC, and ΔADC values. The second group was composed of 22 patients (23.4%) with only staging MR, allowing for the evaluation of pre-ADC values only, while the third group consisted of 20 patients (21.3%) with only restaging MRI, allowing for the evaluation of post-ADC values only. The flow chart in Figure 1 summarizes the study population and subgroups. TRG classes in all subgroups are summarized in Table 2. The mean time interval between staging and restaging MRI was 5 months (range 4–6).

### 3.2. Pre-ADC Values Analysis

**Figure 2:**
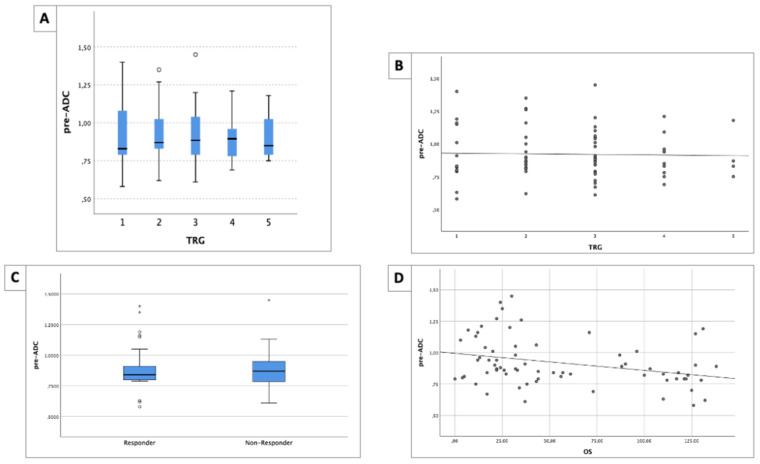
(**A**) Boxplot of pre-ADC values and different TRG classes: no significant difference was found (*p* = 0.930). (**B**) No correlation between pre-ADC values and different TRG classes was found (r = 0.005, *p* = 0.484). (**C**) Boxplot of pre-ADC values between responders and non-responders. No significant difference was found (*p* = 0.796). (**D**) A good negative correlation between pre-ADC values and OS time was found (r = −0.381, *p* = 0.001). OS is expressed in months.

No significant differences in the pre-ADC values among the different TRG classes (*p* = 0.930) [Figure 2A], nor correlations (r = 0.005, *p* = 0.484), were found [Figure 2B].

The mean pre-ADC value of the responders was 0.931 mm^2^/s (±0.195), while, for non-responders, it was 0.896 mm^2^/s (±0.171), with no statistically significant difference between the two groups (*p* = 0.796) [Figure 2C].

A pre-ADC value between responders and non-responders of 0.855 mm^2^/s showed a sensitivity of 44.8%, a specificity of 43.5%, and an AUROC of 47.9% (95% CI 31.6–64.2).

The pre-ADC values showed a significant negative correlation with OS (r = −0.381, *p* = 0.001) [Figure 2D]. The pre-ADC values are summarized in Table 3.

### 3.3. Post-ADC Values Analysis

**Figure 3:**
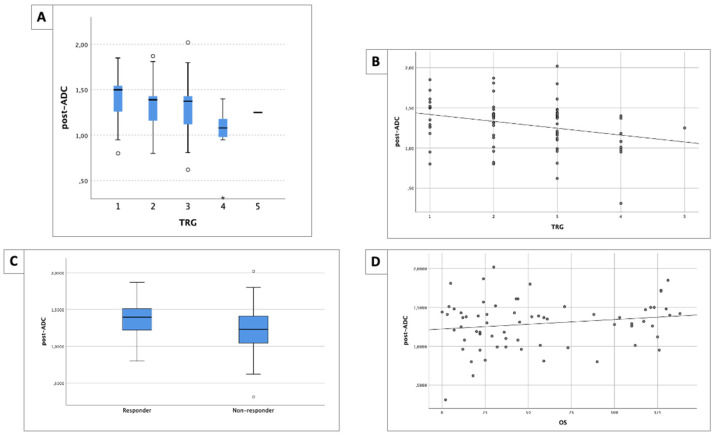
(**A**) Boxplot of post-ADC values and different TRG classes. No significant difference was found (*p* = 0.068). (**B**) A good negative correlation between post-ADC values and TRG classes was found (r = −0.285, *p* = 0.002). (**C**) Boxplot of post-ADC values between responders and non-responders. A significant difference was found (*p* = 0.019). (**D**) No correlation between post-ADC values and OS time was found (r = 0.123, *p* = 0.202). OS is expressed in months.

No significant difference between post-ADC values and each TRG class was found (*p* = 0.068) [Figure 3A]; however, a significative negative correlation was appreciable (r = −0.285, *p* = 0.002) [Figure 3B].

The mean post-ADC value of responders was 1.357 mm^2^/s (±0.279), while, for non-responders, it was 1.229 mm^2^/s (±0.311), with a statistically significant difference (*p* = 0.019) [Figure 3C].

A post-ADC value between responders and non-responders of 1.170 mm^2^/s showed a sensitivity of 77.8%, a specificity of 49.1%, and an AUROC of 63.3% (95% CI 50.4–76.3). The post-ADC values showed no correlation with OS (r = 0.123, *p* = 0.202) [Figure 3D].

The post-ADC values are summarized in Table 3.

### 3.4. ΔADC Analysis

**Figure 4:**
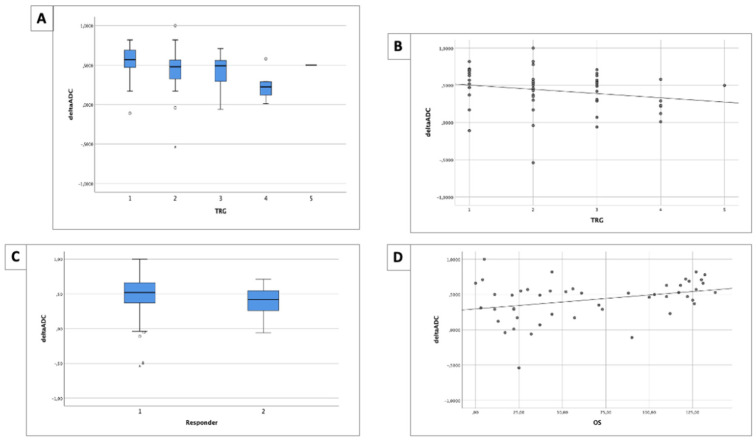
(**A**) Boxplot of ΔADC values and different TRG classes. No significant difference was found (*p* = 0.198). (**B**) A good negative correlation between ΔADC values and TRG classes was found (r = −0.290, *p* = 0.019). (**C**) Boxplot of ΔADC values between responders and non-responders. No significant difference was found (*p* = 0.117). (**D**) A good positive correlation between ΔADC values and TRG classes was found (r = 0.323, *p* = 0.013). OS is expressed in months.

No statistically significant difference between mean ΔADC values and each TRG class (*p* = 0.198) was found [Figure 4A], while a negative correlation was appreciable (r = −0.290, *p* = 0.019) [Figure 4B].

The mean ΔADC value of responders was 0.465 mm^2^/s (±0.279), while, for non-responders, it was 0.378 mm^2^/s (±0.311), with no significant difference between the two groups (*p* = 0.117) [Figure 4C].

A ΔADC value between responders and non-responders of 0.305 mm^2^/s showed a sensitivity of 79.3%, a specificity of 58.2%, and an AUROC of 62.7% (95% CI 47.5–78.0).

The ΔADC values demonstrated a significant positive correlation with mean OS time (r = 0.323, *p* = 0.013) [Figure 4D].

The ΔADC values are summarized in Table 3.

### 3.5. Follow-Up

Four patients (4.2%) were lost to follow-up immediately after surgical intervention; therefore, no data regarding the OS were available, while 12 more patients (12.8%) were lost during the follow-up. During the follow-up period, 24 patients (30.8%) developed a recurrence.

Seven patients (8.9%) died due to the primary disease, while 2 (2.5%) died for different causes.

A total of 29 patients (55.8%) from the first subgroup, five (22.7%) from the second subgroup, and seven patients (35.0%) from the third subgroups were considered responders [Table 2].

Overall, median and mean OS time of responders were 100 (4–138) months and 81 (±46) months, respectively, while, for non-responders, these were 37 (2–130) months and 49 (±42) months, respectively, with a statistically significant difference between the two groups (*p* = 0.019) [Table 3].

A statistically significant difference between TRG classes and mean OS time was found (*p* = 0.038), with a negative correlation (r = −0.374, *p* = 0.004).

## 4. Discussion

Since the “watch-and-wait” strategy could become a valuable alternative to the “traditional” approach of neoadjuvant therapy followed by surgery, the potential predictive role of ADC values in OS could be fundamental in the management of advanced-stage rectal cancer. According to Habr-Gama and her study group [5], the “wait and see” approach could be applied to any patient who shows a complete response to the neoadjuvant treatment. According to some authors [6,11,12], the prognosis of patients undergoing this new approach is the same as those who are surgically treated, but their quality of life is significantly improved. The main limitation of this new conservative strategy is the lack of instruments that could predict patients’ responses with the same accuracy of the pathological examination.

Jacobs et al. [13] found a correlation between pre-CRT ADC values and responses to treatment, with significantly lower pre-ADC values in the good response group compared to the moderate/poor response group, while Monguzzi et al. [14] found no correlation between the pre-ADC values and the response to treatment.

In our study, we found no relationship between the pre-ADC values and TRG class, contrarily to previous studies [13,15,16,17] but in agreement with other authors [14,18]. Considering the pre- and post-treatment variation in ADC values, Cai et al. [19] found that the tumor pre-treatment ADC values in good responders tended to decrease until the second week of CRT, after which they increased again until the end of the therapy.

Sun et al. [20] found a significant difference among pre-, post-, and ΔADC values between responders and non-responders: pre-ADC values were lower in the downstaged group, with downstaging being associated with higher post-ADC values and higher ΔADC values among the responders and a significant difference in the mean percentage change in ADC and mean tumor volume reduction.

Considering only the post-ADC values, although no significant differences could be found between each TRG class and post-ADC values, there was a significant negative correlation between post-ADC values and TRG classes: higher post-ADC values were associated with the lower TRG classes, in line with other authors’ findings [13,14,15,18,21].

Moreover, when patients were divided into responders (TRG 1–2) and non-responders (TRG 3–5), a statistically significant difference was found with respect to post-ADC values.

The ΔADC values were found to be related to treatment response, with a good negative correlation between ΔADC values and TRG classes. In our study, the ΔADC values could be a good predictor of treatment response, because ΔADC tends to increase in patients who present a good response to therapy and are associated with a lower TRG class.

Concerning the prognosis of patients with LARC, Smith et al. [12] stated that patients who underwent surgery had a 3-year DFS rate equal to 73–79%, while OS accounted for 90–92% of patients in the same time interval. In this setting, Curvo-Semedo et al. [22] found that lower pre-treatment ADC values are associated with MRF involvement, nodal involvement, and poorly differentiated tumors, but not with survival.

We found that pre-ADC values have a negative correlation with OS, although the value distribution results were wide. It seems that patients with lower pre-ADC values tend to have a better OS. When evaluating the post-ADC values, no significant difference nor correlation could be found with respect to OS. In our study, post-ADC values correlated better with TRG classes but worse with OS than pre-ADC and ΔADC values.

Moreover, the difference between TRG classes and OS was statistically significant, with a better performance when dividing the patients into responder and non-responder groups.

Interestingly, the main result of this study is that ΔADC values better related to OS and to TRG classes: as the ΔADC increased, treatment response and outcome improved. The evaluation of post-ADC values appears to be important because of ΔADC calculation in the OS assessment and the correlation with TRG class in the treatment response after pathological evaluation.

Only Moon et al. [23] evaluated the complementary value of pre-treatment ADC for predicting tumor recurrence, finding a significant difference in tumor ADC between patients with and without local recurrence, showing that the lower pre-ADC values were associated with a better prognosis.

Bakke et al. [24] performed a small prospective trial which resulted in the inability of ADC values to predict the progression-free survival at any time point, similarly to the results from the perfusion fraction analysis.

The main limitation of ADC values is their variability, which reflects tumor heterogeneity: the presence of necrotic areas inside the primary lesion may account for higher pre-ADC values, lower post-ADC values, and also a certain resistance to radio- and chemotherapy [19,25].

One limitation of this study is that no precise correlation between the imaging data and the surgically resected specimen was possible: this could have made the comparison of the mean ADC values and the TRG classes difficult, especially in relation to post-treatment examinations where the tumor delineation results are particularly challenging due to post-RT fibrosis and tumor volume reduction. Another limitation of this study could be the ROI delineation. According to Blazic et al. [26], the variations in ROI number, size, and position substantially influence tumor ADC measurements and, in turn, the correlation with the TRG grade. Plus, artifacts (such as intraluminal air) may have negatively affected the ROI positioning and, thus, the ADC values calculation. Moreover, the mean interval among the end of CRT, restaging MRI, and surgery was variable among patients: this may have influenced the ADC measurements, considering that differences in tissue fibrotic replacement could lead to heterogeneous results. Finally, only one radiologist manually drew ROIs on pathological tissues; consequently, reliability analysis could not be computed.

Even if promising, our results should be applied to larger cohorts of patients, from different centers, to determine the potentiality of ADC values before and after CRT. In this setting, considering the raising interest in quantitative imaging, future directions should be also focused on the importance of quantitative DWI values in LARC, mainly applying radiomics signatures. Robust results can be achieved with collaborations between expert and non-expert centers, reducing the risk of bias and increasing the number of enrolled patients who undergo standard MR protocols.

## 5. Conclusions

The pre-ADC and ΔADC values could become useful predictors of patient prognosis, thus helping the treatment planning. On the other hand, the post-ADC values, thanks to their relationship with the TRG classes, could be the ideal tool to predict histopathological responses and plan a conservative approach for the treatment of rectal cancer; however, a threshold post-ADC value to clearly distinguish between responders and non-responders has yet to be found.

## Figures and Tables

**Table 1:** MR protocol. Ax: axial, Sag: sagittal, Parax: paraxial, orthogonal to the long axis of the tumor, Paracor: paracoronal, parallel to the long axis of the tumor. * SENSE factor: 1.5.

	Ax TSE_T1W	Sag TSE_T2W	Parax TSE_T2W	Paracor TSE_T2W	Ax DWI
Thickness (mm)	3	3.5	3	3	6
Slices (n)	20	18	20	20	12
Gap (mm)	3	3.5	0.5	0.5	6
TR (ms)	612	4570	5058	5058	3000
TE (ms)	14	120	125	125	74
Flip Angle (°)	90	90	90	90	90
FOV	180 × 85	180 × 85	180 × 100	180 × 100	380 × 80
Matrix	272 × 320	256 × 256	256 × 256	256 × 256	240 × 256
NSA (n)	4	4	4	4	4
Time (min)	4.43	3.05	3.47	3.47	1.30 *

**Table 2:** Summary of the entire cohort and subgroup division. Group 1: patients with MRI pre- and post-CRT. Group 2: patients with MRI only pre-CRT. Group 3: patients with MRI only post-CRT. Responders: patients with TRG 1 and 2. Non-responders: patients with TRG 3, 4, 5.

N = 94		Group			Total	*p*-Value
		1	2	3		
TRG (n; %)	1	13; (25.0)	2; (9)	2; (10.0)	17; (18.2)	0.071
	2	16; (30.8)	3; (13.7)	5; (25.0)	24; (25.5)	
	3	16; (30.8)	10; (45.4)	10; (50.0)	36; (38.3)	
	4	6; (11.4)	4; (18.2)	3; (15.0)	13; (13.8)	
	5	1; (2%)	3; (13.7)	0; (0.0)	4; (4.2)	
Responder (n; %)		29; (55.8)	5 (22.7)	7; (35.0)	36; (50.0)	0.285
Non-responder (n; %)		23; (44.2)	17 (77.3)	13; (65.0)	36; (50.0)	
Total		52; (55.3)	22; (23.4)	20; (21.3)	94; (100)	

**Table 3:** ADC values of the entire cohort according to TRG classes and ADC values between responders and non-responders. All ADC values are expressed in mm^2^/s as mean ± SD. A *p*-value < 0.05 was considered statistically significant (bold values).

N = 94		Pre-ADC	*p*-Value	Post-ADC	*p*-Value	ΔADC	*p*-Value	OS (Months)		*p*-Value
								Mean (±SD)	Median (Min–Max)	
TRG	1	**0.912 ± 0.223**	**0.930**	**1.391 ± 0.278**	**0.068**	**0.515 ± 0.254**	**0.198**	92 (±44)	110 (4–131)	**0.038**
	2	0.944 ± 0.191		1.333 ± 0.284		0.424 ± 0.357		71 (±48)	64 (5–138)	
	3	0.919 ± 0.182		1.294 ± 0.294		0.424 ± 0.211		50 (±44)	37 (2–130)	
	4	0.904 ± 0.158		1.040 ± 0.317		0.241 ± 0.193		53 (±42)	44 (13–112)	
	5	0.907 ± 0.188		1.250		0.500		11	11 (11)	
Responder		0.931 ± 0.195	0.796	1.358 ± 0.279	**0.019**	0.465 ± 0.313	0.117	81 (±46)	100 (4–138)	**0.019**
Non-Responder		0.896 ± 0.171		1.229 ± 0.312		0.378 ± 0.215		49 (±42)	37 (2–130)	

## Data Availability

Data are available upon request.
